# Distinction between allergic rhinitis alone and allergic rhinitis + asthma in MASK-air® real-world studies; a ARIA-WAO task force review^☆^

**DOI:** 10.1016/j.waojou.2026.101435

**Published:** 2026-07-31

**Authors:** Bernardo Sousa-Pinto, Rafael José Vieira, Ana Margarida Pereira, Alkis Togias, Ludger Klimek, Gerard H. Koppelman, Erik Melén, Nikolaos G. Papadopoulos, Maryam Ali Al-Nesf, Luisa Brussino, Alvaro A. Cruz, Bilun Gemicioglu, Violeta Kvedariene, Désirée E. Larenas-Linnemann, Rachel Nadif, Frederico S. Regateiro, Boleslaw Samolinski, Marine Savouré, Luis Taborda-Barata, Sanna K. Toppila-Salmi, Maria Teresa Ventura, Philip W. Rouadi, Alessandro Fiocchi, Maximiliano R. Gomez, Gary W.K. Wong, Ignacio J. Ansotegui, Tari Haahtela, Josep M. Anto, Torsten Zuberbier, Joao A. Fonseca, Mario Morais-Almeida, Jean Bousquet

**Affiliations:** a MEDCIDS - Department of Community Medicine, Information and Health Decision Sciences, Faculty of Medicine, University of Porto, Porto, Portugal; b CINTESIS@RISE– Health Research Network, Faculty of Medicine, University of Porto, Porto, Portugal; c Allergy Unit, Instituto and Hospital CUF, Porto, Portugal; d Division of Allergy, Immunology, and Transplantation (DAIT), National Institute of Allergy and Infectious Diseases, NIH, Bethesda, MD, USA; e Department of Otolaryngology, Head and Neck Surgery, Universitätsmedizin Mainz, Mainz, Germany; f Center for Rhinology and Allergology, Wiesbaden, Germany; g University of Groningen, University Medical Center Groningen, Beatrix Children's Hospital, Department of Pediatric Pulmonology and Pediatric Allergology, Groningen, the Netherlands; h University of Groningen, University Medical Center Groningen, GRIAC Research Institute, Groningen, the Netherlands; i Department of Clinical Science and Education, Södersjukhuset, Karolinska Institutet, Stockholm, Sweden; j Sach's Children and Youth Hospital, Södersjukhuset, Stockholm, Sweden; k Allergy Department, 2nd Pediatric Clinic, University of Athens, Athens, Greece; l Adult Allergy and Immunology Division, Hamad Medical Corporation, Doha, Qatar; m Department of Medical Sciences, University of Torino, Torino, Italy; n Allergy and Clinical Immunology Unit, Mauriziano Hospital, Torino, Italy; o Fundacao ProAR and (Faculdade de Medicina da) Universidade Federal da Bahia, Salvador, Bahia, Brazil; p Department of Pulmonary Diseases, Cerrahpaşa Faculty of Medicine, Istanbul University-Cerrahpaşa, Istanbul, Turkey; q Department of Pulmonary Diseases, Institute of Pulmonology and Tuberculosis, Istanbul University-Cerrahpaşa, Istanbul, Turkey; r Institute of Clinical Medicine, Clinic of Chest Diseases and Allergology, Faculty of Medicine, Vilnius University, Vilnius, Lithuania; s Institute of Biomedical Sciences, Department of Pathology, Faculty of Medicine, Vilnius University, Vilnius, Lithuania; t Center of Excellence in Asthma and Allergy, Médica Sur Clinical Foundation and Hospital, México City, Mexico; u Université Paris-Saclay, UVSQ, Université Paris-Sud, Paris, France; v Inserm, Équipe d'Épidémiologie Respiratoire Intégrative, CESP, Villejuif, France; w Allergy and Clinical Immunology Department, Hospitais da Universidade de Coimbra, Unidade Local de Saúde de Coimbra, Coimbra, Portugal; x Center for Innovative Biomedicine and Biotechnology (CIBB), Faculty of Medicine, University of Coimbra, Coimbra, Portugal; y Institute of Immunology, Faculty of Medicine, University of Coimbra, Coimbra, Portugal; z UBIAir - Clinical & Experimental Lung Centre and CICS-UBI Health Sciences Research Centre, University of Beira Interior, Covilhã, Portugal; aa Department of Prevention of Environmental Hazards, Allergology and Immunology, Medical University of Warsaw, Warsaw, Poland; ab Barcelona Institute for Global Health (ISGlobal), Barcelona, Spain; ac Universitat Pompeu Fabra (UPF), Barcelona, Spain; ad CIBER Epidemiología y Salud Pública (CIBERESP), Barcelona, Spain; ae RISE-Health, Faculty of Health Sciences, and UBIAir-Clinical & Experimental Lung Centre, University of Beira Interior, Covilhã, Portugal; af Department of Immunoallergology, Cova da Beira University Hospital Centre, Covilhã, Portugal; ag Department of Otorhinolaryngology, University of Eastern Finland and the North Savo Wellbeing Services County, Kuopio, Finland; ah Department of Allergy, Skin and Allergy Hospital, Inflammation Center, Helsinki University Hospital and University of Helsinki, Helsinki, Finland; ai University of Bari Medical School, Bari, Italy; aj Institute of Sciences of Food Production, National Research Council (ISPA-CNR), Bari, Italy; ak Department of Otolaryngology, Head and Neck Surgery, Eye and Ear University Hospital, Beirut, Lebanon; al Department of Otorhinolaryngology, Head and Neck Surgery, Dar Al Shifa Hospital, Salmiya, Kuwait; am Allergy Unit, Bambino Gesù Children's Hospital, IRCCS, Rome, Italy; an School of Health Sciences, Catholic University of Salta, Salta, Argentina; ao Department of Paediatrics, Prince of Wales Hospital, Chinese University of Hong Kong, Shatin, Hong Kong, China; ap Department of Allergy and Immunology, Hospital Quironsalud Bizkaia, Bilbao, Spain; aq Skin and Allergy Hospital, Helsinki University Hospital, and University of Helsinki, Helsinki, Finland; ar Institute of Allergology, Charité – Universitätsmedizin Berlin, Corporate Member of Freie Universität Berlin and Humboldt-Universität zu Berlin, Berlin, Germany; as Fraunhofer Institute for Translational Medicine and Pharmacology ITMP, Immunology and Allergology, Berlin, Germany; at Allergy Center, CUF Descobertas Hospital, Lisbon, Portugal; au ARIA (Allergic Rhinitis and Its Impact on Asthma), Montpellier, France

**Keywords:** Asthma, Rhinitis, Conjunctivitis, Multimorbidity, MASK-air, Real-life studies

## Abstract

The recent ARIA-MeDALL hypothesis proposed in 2023 that allergic rhinitis (AR) alone and allergic rhinitis and asthma (A) multimorbidity (AR + A) represent 2 distinct diseases. To improve the knowledge on this topic, we have gathered data from real-world studies using MASK-air®. According to our analyses: (i) An “extreme allergy phenotype” [A + AR + C, Conjunctivitis] was confirmed and found to be more severe (symptoms and work productivity) than single diseases alone. (ii) Patients with AR + A required more rhinitis medications, and had more severe VAS levels for nasal and ocular symptoms than those with AR alone in all countries tested. (iii) In clusters with poorly controlled AR, the frequency of co-medicating with more than 1 rhinitis drug was higher in AR + A than in AR alone. (iv) In the CONSTANCES general population cohort, a co-medication pattern (intranasal corticosteroid and oral H1-antihistamines) was associated with the presence of AR + A (vs AR alone). This co-medication pattern was associated in MASK-air® with a poorer AR control than monotherapy. (v) Patients with AR + A had different EQ-5D patterns than those with AR alone. (vi) Large differences were found between AR alone and AR + A in work impairment, resulting in higher weekly indirect costs in all OECD countries in AR + A by comparison to AR alone. Although mHealth studies are hypothesis-generating, and usually not reliable for testing or confirming hypotheses, our results are strongly in support of the nosologic distinction between A + AR and AR alone.

## Introduction

The recent ARIA-MeDALL hypothesis proposed in 2023 that allergic rhinitis (AR) alone and allergic rhinitis and asthma (AR + A) represent 2 distinct diseases.^1^ Evidence for this hypothesis has been obtained from the MeDALL (Mechanisms of the Development of Allergy) study,^2^ epidemiologic studies^3^, ^4^, ^5^ and from MASK-air® data.^6^ It is grounded on insights from polysensitisation and multimorbidity, advances in mHealth for novel phenotype definition, confirmation in canonical epidemiologic studies, and genomic findings (Table 1 online).

**Table 1:** Summary of results

• The extreme allergy phenotype (A + R + C) was confirmed and found to be more severe than single diseases alone.^14^^,^^16^^,^^18^ • Patients with AR + A required more rhinitis medications, and had more severe VAS levels for nasal and ocular symptoms than those with AR alone.^18^ • Clusters with poorly controlled rhinitis^17^ displayed a higher frequency of rhinitis co-medication in AR + A than in AR^16^ and had a higher use of rhinitis co-medication.^16^ • In the CONSTANCES general population cohort, AR + A participants were less well controlled than those with AR alone.^22^^,^^23^ These features were associated with poor control in the MASK-air study.^21^ • Similar A + R patterns were found in the ARIA classification in CONSTANCES and MASK-air.^24^ • Overall, a co-medication pattern was associated with a poorer AR control than monotherapy.^21^ • Reduced control of asthma and need for co-medication in rhinitis were found in uncontrolled asthmatics (AR + A) by comparison to AR alone.^15^ • Patients with AR + A had different EQ-5D patterns than those with AR alone.^6^ • Large differences were found between R alone and R + A in work impairment.^34^ • Weekly indirect costs in all countries of OECD are increased in AR + A by comparison to AR alone.^36^

To improve the knowledge on these apparently distinct conditions, more studies, including real-world studies, are needed. MASK-air® is a large observational implementation study in which an mHealth app has been used by over 75,000 people with AR and/or A in 35 countries.^7^ MASK-air® reports real-life data and is a Good Practice of the Directorate General Health and Food Safety (EU)^8^ and of Organisation for Economic Co-operation and Development (OECD)^9^ on digitally enabled, patient-centred care. In MASK-air®, very few patients have A without AR, and A alone could not be studied.^10^

A World Allergy Organization (WAO) Task Force on the distinction between rhinitis and asthma phenotypes was established in March 2025, and this is the first paper. The aim of this manuscript is to critically review the evidence on the occurrence, severity and impact of AR + A compared to AR alone using MASK-air® data.

## 1-“Extreme allergy phenotype”

In the pooled birth cohort MeDALL, the coexistence of A, AR, and atopic dermatitis (AD) in the same child is rare but more common than expected by chance, and independent of IgE sensitisation.^11^^,^^12^ Patients with A + AR + AD had more severe individual diseases than those with A and/or AR, proposing the concept of an “extreme allergy phenotype”. These data suggest that these diseases share causal mechanisms of multimorbidity beyond IgE sensitisation and that there is a rare distinct group of children with all 3 disorders (the extreme allergy phenotype: A + AR + AD).

This study was confirmed by a meta-analysis on data from ISAAC (International Study of Asthma and Allergies in Childhood, 31 studies in 1,430,329 children in 102 countries).^13^ Only a minority of children suffer from all 3 disorders (A + AR + AD) but this co-occurrence is significantly higher than expected by chance.

**Fig. 1:**
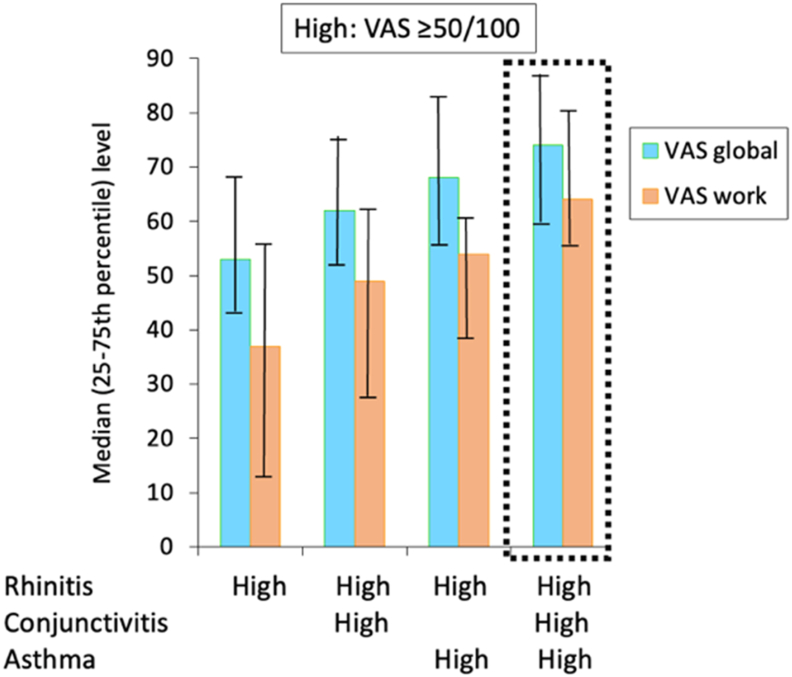
**Impact on work and VAS global depending on the RCA multimorbidity** (from Bousquet et al^14^).

MASK-air® cross-sectional and longitudinal studies have helped to refine the “extreme allergy phenotype” concept. 14, 15, 16 MASK-air® data in adolescents and adults showed the same findings with A, AR and conjunctivitis (C). 14, 15, 16 AD was not examined. In one of the first cross-sectional MASK-air® studies, 32,585 days of 4210 patients with high levels (VAS>50/100) of AR, C and/or A were compared.^14^ A novel “Rhinitis High - Asthma High - Conjunctivitis High” pattern was identified in 2.9% days. It was particularly different from the remainder on uncontrolled VAS global^17^ and impaired work productivity (Fig. 1).

These results were confirmed in 2 other studies (1 cross-sectional and 1 longitudinal) in a large number of subjects which showed that the daily patterns were confirmed in patients clustering either A or AR.^16^^,^^18^ In particular, disease control was worse in patients with A + AR + C. These MASK-air® data support the ARIA-MeDALL hypothesis on allergic rhinitis phenotypes.

## MASK-air® data strengthening the ARIA-MeDALL hypothesis on allergic rhinitis phenotypes

### Cross-sectional study

**Fig. 2:**
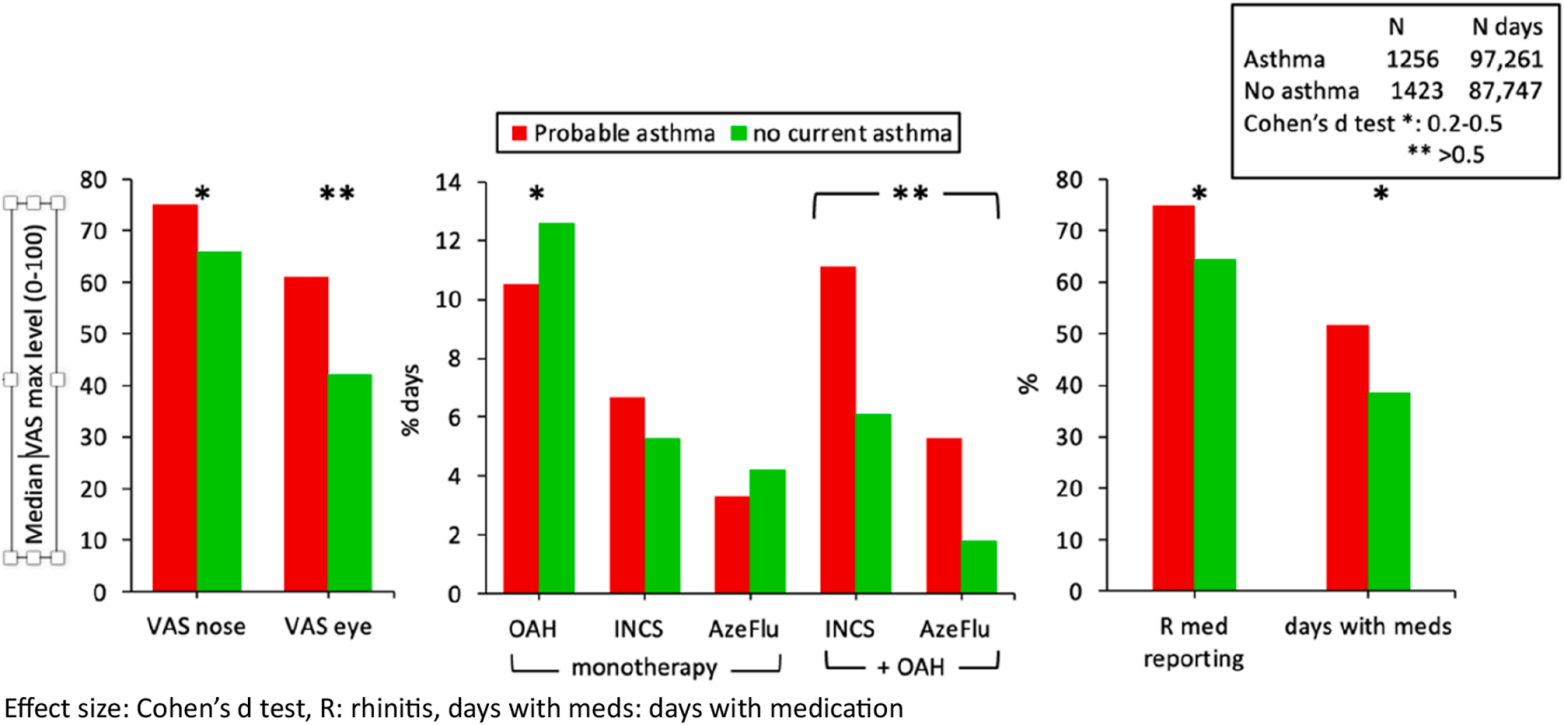
**Differences between patients with asthma + rhinitis (probable asthma [Bousquet et al**^**15**^]**) and rhinitis** (from Bousquet et al^18^).

A cross-sectional study was carried out on 3797 patients (256,839 days). Probable asthma was defined according to a study using cluster analysis of patients with asthma and nasal symptoms using the MASK-air® app.^15^ By comparison to AR, those with AR + A (i) had more days under co-medication, and (ii) had more severe VAS levels for nasal and ocular symptoms and work (Fig. 2).^18^

**Fig. 3:**
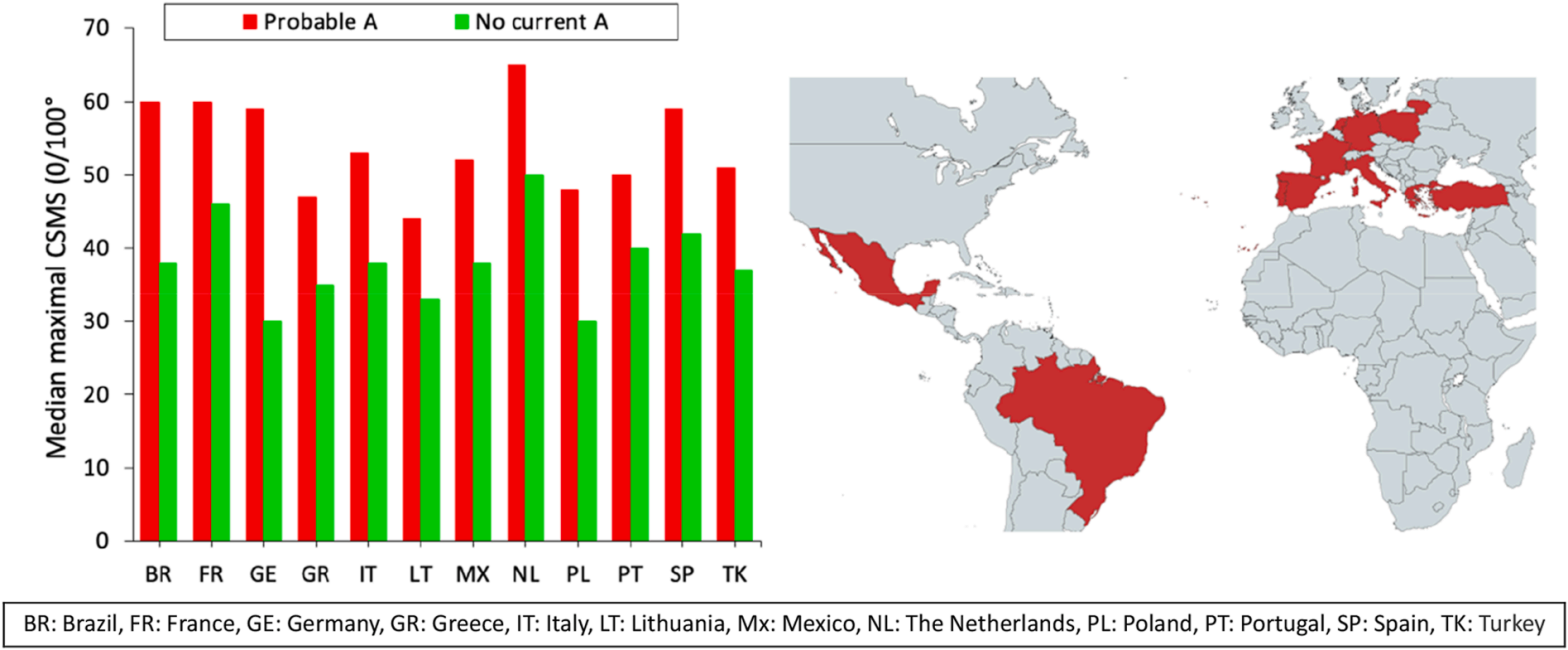
**Combined Symptom-Medication Score (CSMS) in no current or probable asthma in 12 countries** (from Bousquet et al^18^).

More robust evidence for the above concept was obtained (i) when assessing data in the sub-analysis of 282 patients enrolled by physicians and (ii) by observing similar results in the different countries of the study (Fig. 3) indicating that the A + R and the R alone phenotypes are similar in Northern and Southern Europe, Turkiye, Mexico, and Brazil.

### Longitudinal study

In MASK-air®, data have frequently been analysed cross-sectionally. We also analysed data longitudinally, focusing on the weeks for which patients had answered a rhinitis daily questionnaire on all 7 days. We used k-means clustering algorithms to define clusters of weeks according to the trajectories of reported daily rhinitis symptoms. We analysed 113,239 days (16,177 complete weeks) from 2590 patients. Clusters with poorly controlled rhinitis^17^ displayed a higher frequency of rhinitis co-medication and AR + A as compared to clusters with well-controlled AR.^16^

### Studies using the ARIA classification

**Fig. 4:**
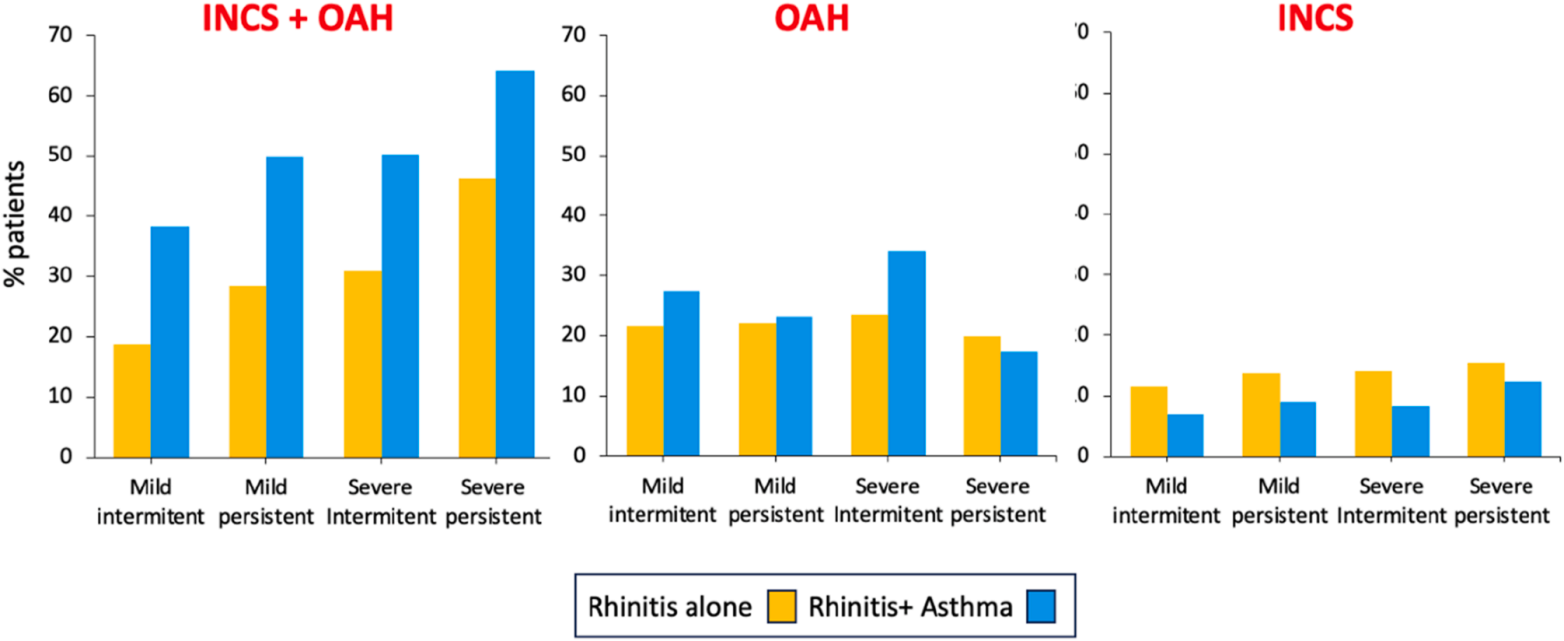
**Medications reported in patients with rhinitis in the CONSTANCES study according to ARIA classification and the impact of asthma (AR + A)** (from Savoure et al^20^).

**Fig. 5:**
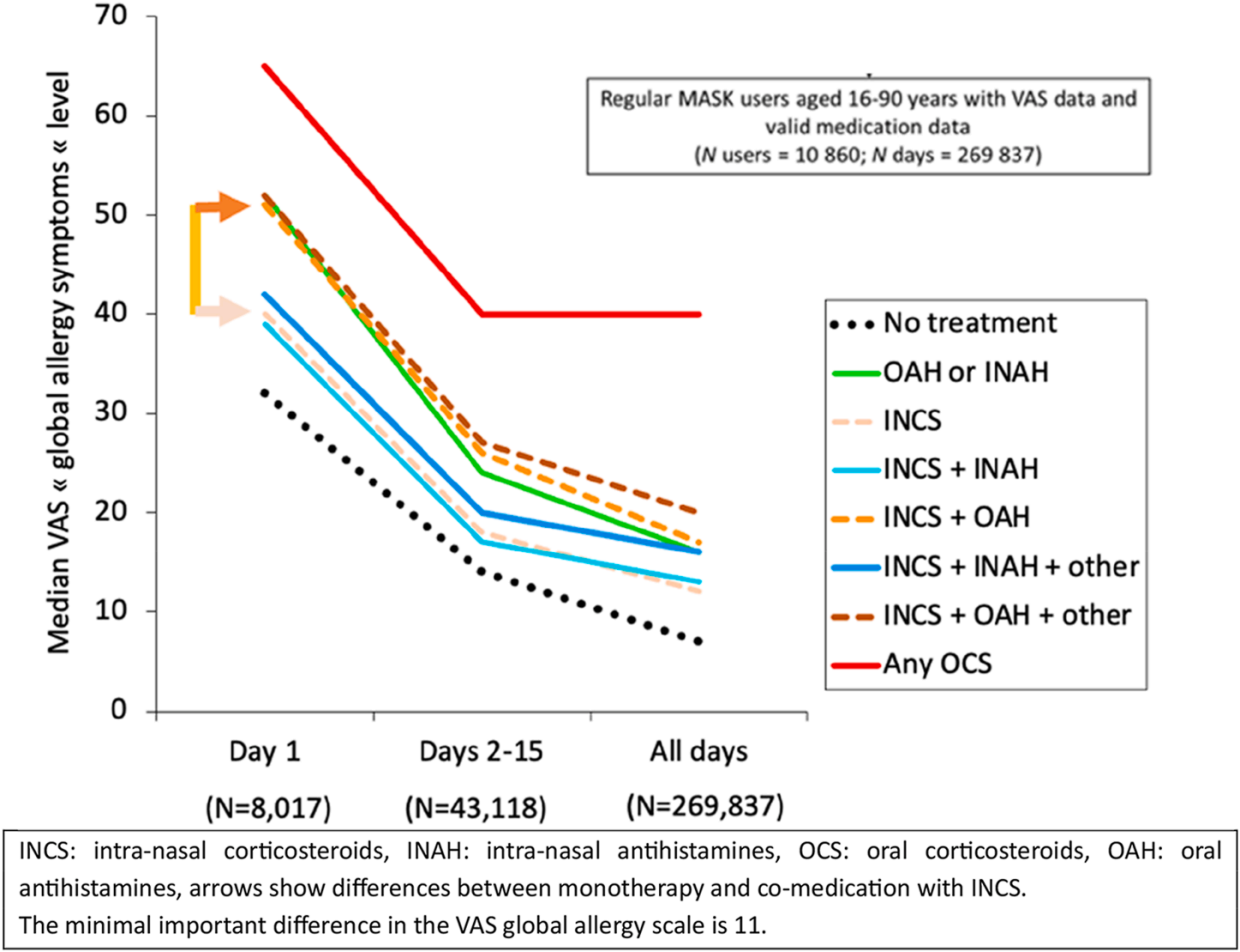
**Median global allergy VAS depending on medications in a MASK-air® cross-sectional study** (from Sousa-Pinto et al^21^).

CONSTANCES is a general-purpose population-based epidemiological cohort in France. It is a nationally representative sample.^19^ In the second CONSTANCES study in AR, for the 4 ARIA classes, participants with AR + A more often reported a treatment with intranasal corticosteroids and oral antihistamines than those with AR alone (Fig. 4).^20^ MASK-air® data offer a possible interpretation of these CONSTANCES findings. In MASK-air®, a co-medication pattern was associated with a poorer AR control than monotherapy (the fixed combination azelastine-fluticasone was considered as monotherapy) (Fig. 5),^21^ suggesting that AR + A participants of the CONSTANCES cohort were less well controlled than those with AR alone.^22^^,^^23^

**Fig. 6:**
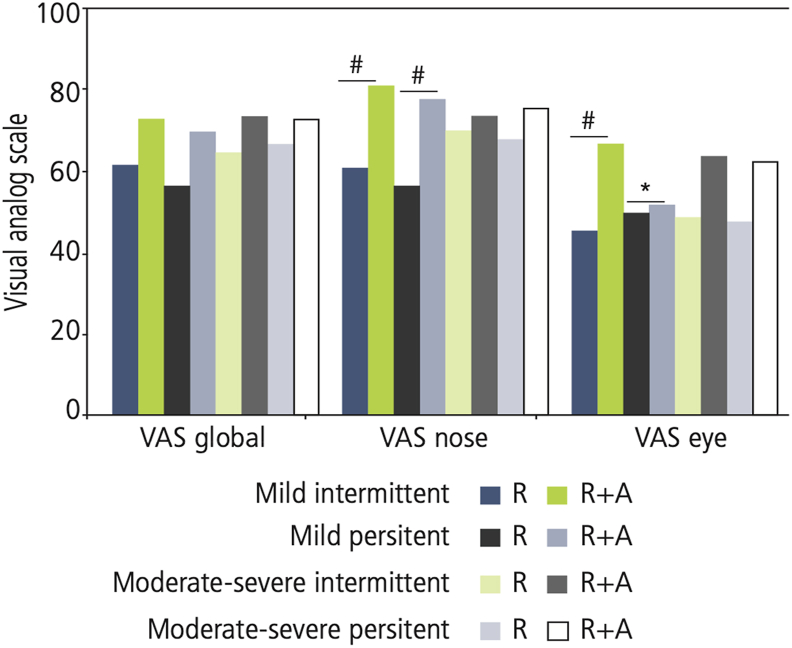
**Global visual analogue scale in AR and AR + A depending on ARIA classes obtained by MASK-air®** (from Sousa-Pinto et al^24^).

In a cross-sectional study based on 2273 MASK-air® users (180,796 days), we assessed the distribution of ARIA classes. Within most ARIA classes, particularly those of mild rhinitis, VAS nose and VAS eye scores were higher in patients with AR + A compared to those with AR alone (Fig. 6).^24^ These results are in accordance with those of the CONSTANCES study.^20^ It is also important that VAS eye and VAS nose levels followed the same pattern, which is compatible with the notion of allergic multimorbidity.

### Confirmation of MASK-air® data in canonical epidemiologic studies

The results of mHealth apps are hypothesis-generating and need to be confirmed in canonical epidemiologic studies.^1^ Several such studies have shown that (i) the risk of adult-onset A increases with the number of allergic multimorbidities,^25^ (ii) severe A is associated with multimorbidity,^26^ (iii) blood eosinophil levels are increased in AR + A by comparison to AR, 3, 4, 5 (iv) patients with AR + A have an earlier onset of symptoms and a stronger history of parental allergy than those with A alone,^27^^,^^28^ and (v) at puberty, sex hormones impact the male/female allergy ratio, particularly in multimorbidity.^29^^,^^30^ These results also accord with those of the CONSTANCES study on rhinitis phenotypes.^3^

## Control of asthma and allergic rhinitis phenotypes

**Fig. 7:**
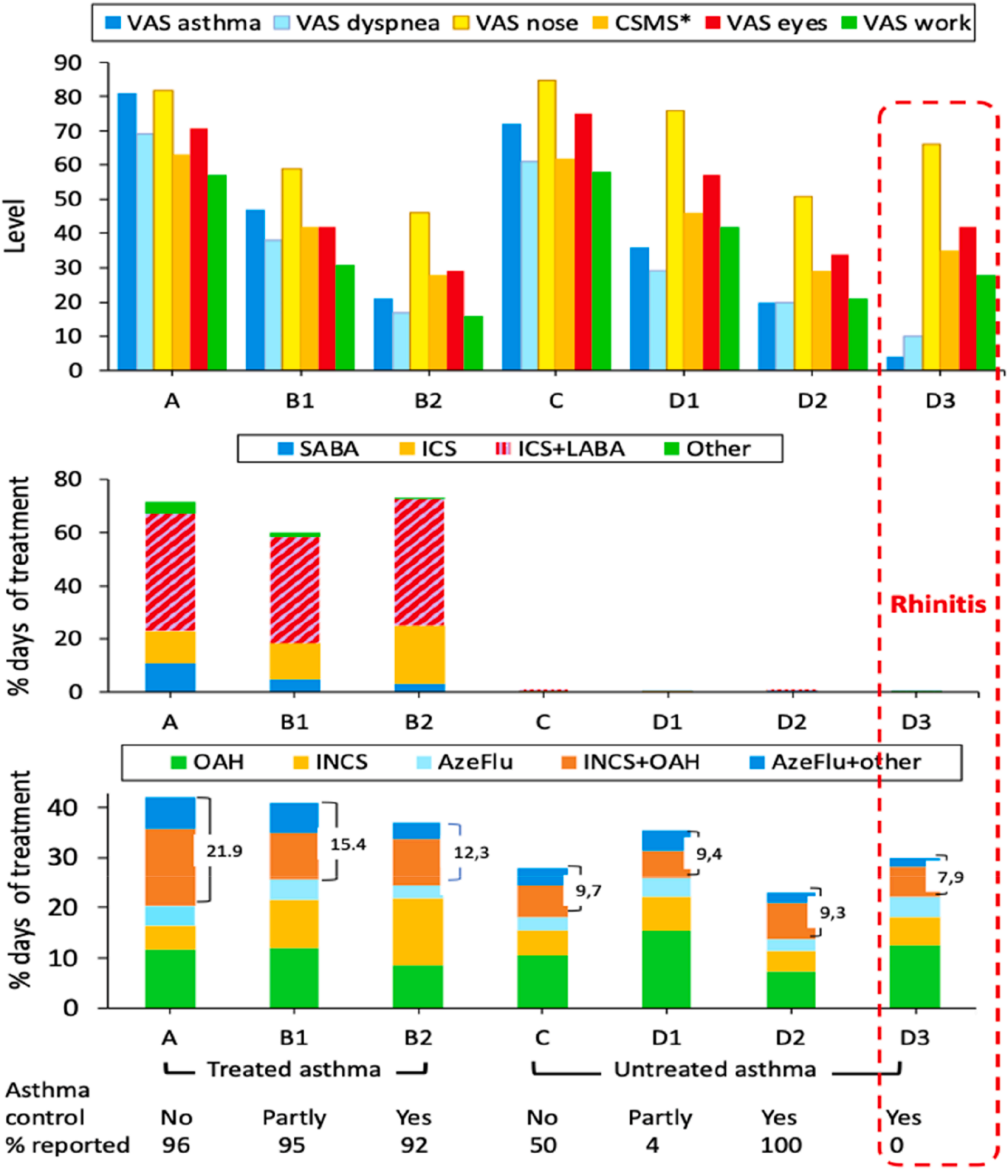
**Rhinitis treatment in patients with treated or untreated asthma or rhinitis** (from Bousquet et al^15^).

In 8075 MASK-air® users,^15^ k-means cluster analysis methods identified 7 groups of A symptom and treatment patterns. One of these clusters (cluster D3) had minimal asthma symptoms and no asthma treatment, representing primarily patients with AR alone. Patients with treated A needed more rhinitis treatment with OAH and INCS or AzeFlu combination (12.3%–21.9%) than those with untreated asthma (9.3%–9.7%) or, most importantly, those with AR alone (7.9%) (Fig. 7).

## Relationship between phenotype and impact (QOL, activities, work, education)

### Impact of control on EQ-5D-5L utilities and VAS

**Fig. 8:**
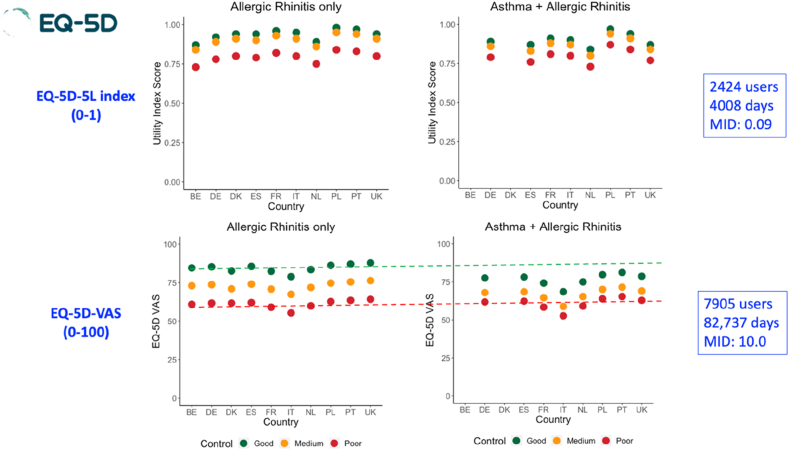
**Differences in utilities and EQ-5D VAS levels associated with uncontrolled nose, eye and asthma symptoms in European countries** (from^33^).

EQ-5D is a generic instrument used to evaluate health-related quality of life (HRQOL). The EQ-5D descriptive system is a preference-based HRQL measure with 1 question for each of the 5 dimensions that include mobility, self-care, usual activities, pain/discomfort, and anxiety/depression.^31^^,^^32^ EQ-5D also includes a VAS on the global health status. In a cross-sectional study of European users, utilities computed based on EQ-5D-5L and EQ-5D VAS were compared for different disease control levels. Spearman rank test coefficients assessed the correlation between (i) the EQ-5D utility index score or the EQ-5D VAS and (ii) symptom VASs (eye, nose, and asthma) (Fig. 8).^33^ There were differences between AR alone and AR + A, with lower mean utilities and mean EQ-5D VAS scores observed for AR + A compared with AR alone.

For the EQ-5D-5L index, good and medium controlled symptoms are better differentiated in AR alone than in AR + A. For the VAS, differences higher than the MID were only seen between controlled and poorly controlled symptoms in AR alone.

### Work impairment and weekly indirect costs per country

**Fig. 9:**
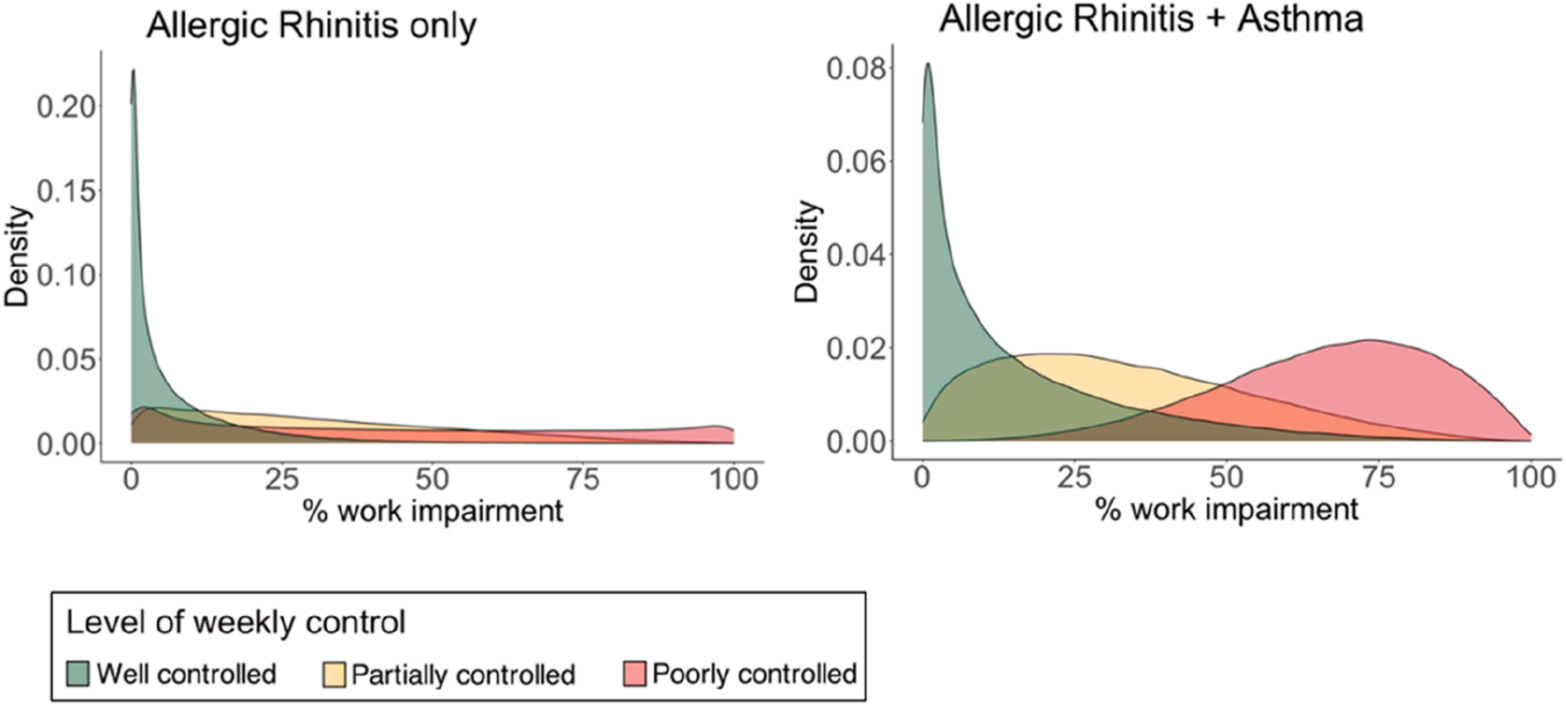
**Total percent work impairment in patients with allergic rhinitis per level of rhinitis control** (from^34^).

A cross-sectional study using the Work Productivity and Activity Impairment Allergic Specific (WPAI-AS) questionnaire measured the impact of AR on work productivity (presenteeism and absenteeism) depending on rhinitis control.^34^ Large differences were found between AR alone and AR + A, with a higher level of work impairment in individuals with AR + A (Fig. 9).

**Fig. 10:**
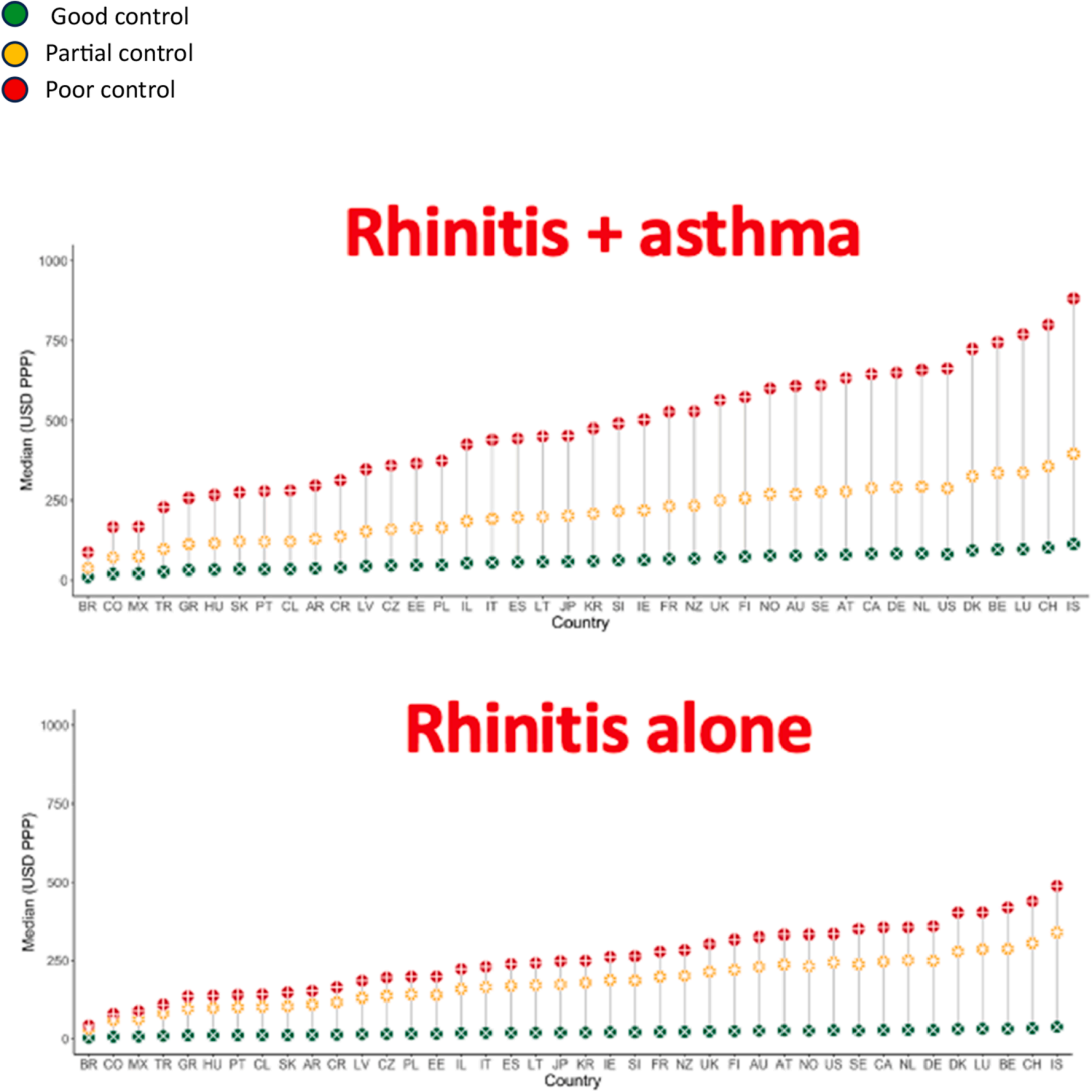
**Median weekly costs of allergic rhinitis due to total work impairment (absenteeism and presenteeism) per country and level of rhinitis control for all patients with allergic rhinitis alone (A), and allergic rhinitis + asthma (B).** (From Vieira et al^36^).

The monetary impact (indirect costs) of AR was assessed based on its impact on work productivity for all OECD countries and for countries where MASK-air® is available. The human-capital method was used wherein productivity is measured by the wages received by a worker.^35^ Patients with AR + A showed higher overall work impairment than patients with AR alone, particularly in poorly controlled weeks (median work impairment in AR alone = 39.1% [P25–P75 = 12.5–71.9%]; median work impairment in AR + A = 68.4% [P25–P75 = 54.6–80.2%]). Total weekly costs (accounting for absenteeism and presenteeism) in poorly controlled weeks of patients with AR alone ranged from 42.5 US$ purchase power parities (PPP; P25–P75 12.3–112.3 US$ PPP) in Brazil to 488.1 US$ PPP (P25–P75 156.8–961.6 US$ PPP) in Iceland. For patients with AR + A, the total costs ranged from 87.3 US$ PPP (P25–P75 42.7–177.5 US$ PPP) in Brazil to 882.5 US$ PPP (P25–P75 595.9–1282.7 US$ PPP) in Iceland (Fig. 10).^36^

## MASK-air® data in conjunctivitis multimorbidity

Differences between AR alone and AR + C were identified with MASK-air® data^14^^,^^27^^,^^37^^,^^38^ and confirmed in canonical epidemiologic studies. These studies have shown that ocular symptoms (i) are more common in AR + A than in AR alone,^38^ (ii) are associated with the severity of nasal symptoms,^39^^,^^40^ and (iii) are important to consider in severe asthma.^39^ These data indicate that C should be considered as a separate disease in AR or AR + A and should be embedded in the multimorbid phenotype.

## Limitations

The results reported herein derive from a single set of studies using an app. To our knowledge, there is no replication with any other app. Moreover, there may be selection bias. However, all data point to the distinction between AR and AR + A. These data are reproducible in all countries tested, and the sample size is extremely large.

mHealth apps can provide large quantities of data that can primarily be used in cross-sectional, hypothesis-generating studies. However, we have confirmed most of the mHealth-generated data with bona fide epidemiologic studies, strengthening their significance. On the other hand, it is important to note that causality cannot be inferenced using the MASK-air® data.

Also, due to privacy concerns and GDPR regulations, no children data can be obtained with mHealth apps. However, birth cohort studies have confirmed MASK-air data from adults in paediatric populations.

## Interpretation

The 9 MASK-air® studies support the ARIA-MeDALL hypothesis that AR alone and AR + A represent 2 distinct conditions. There are 2 important studies that assessed genomics in R and R + A. A discovery study in MeDALL (birth cohorts in Europe, transcriptomics and RT-PCR) and a validation study in EVA-PR (birth cohort in Puerto-Rico, RNA sequencing) yielded the same results: Multimorbidity (A, R, AD) was associated with T2 signalling genes including *IL-5* (eosinophils) and *IL-33* (polysensitisation and eosinophilia).^41^ In MeDALL, R-specific genes have been identified and were mostly associated with TLR (Toll-Like Receptors) signalling pathways and *IL-17*.^41^^,^^42^ A large study identified A endotypes in 3 paediatric cohorts by using transcriptomics in nasal epithelium.^43^ Nasal transcriptomic profiles were consistent with T2^HIGH^, T17 ^HIGH^, and T2^LOW^/T17^LOW^ endotypes. Although this study did not consider R, it showed the importance of T2 ad Th17.

## Conclusions

(Table 1). However, (i) more genetic and epigenetic data are needed to understand whether there are 2 completely distinct diseases or a switch between the 2 pathways, (ii) there is a need for a study combining all MASK-air® data to better appreciate the effect size of the differences between the 2 diseases, and (iii) an estimation is required of the potential benefit in guiding choices in guideline development and in clinical practice.

## Authors’ consent for publication

All authors agree to the publication of this manuscript.

## Authors' contributions

JB, MMA and BSP participated in writing the manuscript. All the other authors participated in the critical revision and editing of the manuscript.

## Availability of data and materials

Not applicable. This is a review manuscript.

## Ethics approval

Not applicable. This is a review manuscript.

## Disclosure statement regarding use of artificial intelligence technology tools

Regarding the use of generative artificial intelligence (AI) and AI-assisted technologies, we have nothing to disclose.

## Disclaimer

Dr. Alkis Togias’ co-authorship of this report does not constitute endorsement by the National Institute of Allergy and Infectious Diseases, the National Institutes of Health or any other Agency of the United States Government.

## Funding sources

ARIA (Allergic Rhinitis and its Impact on Asthma), Montpellier, France; MASK-air SAS, Montpellier, France; CINTESIS@RISE– Health Research Network, Porto, Portugal.

## Conflicts of interest statement

J. Bousquet reports personal fees from Cipla, Menarini, Mylan, Novartis, Purina, Sanofi-Aventis, Teva, Noucor, other from KYomed-Innov, other from Mask-air-SAS, outside the submitted work.

N. Papadopoulos reports grants from Nestle, Numil, Vianex, Vibrant, personal fees from Abbott, Astra Zeneca, GSK, HAL, Menarini, Novartis, Danone Nutricia, OM Pharma, Regeneron, Sanofi, outside the submitted work.

L. Klimek reports personal fees from Allergopharma, personal fees from Viatris, personal fees from LETI Pharma, personal fees from Stallergenes, personal fees from ALK Abelló, personal fees from HAL Allergie, personal fees from Sanofi, personal fees from AstraZeneca, personal fees from GSK, personal fees from Inmunotek, personal fees from Regeneron Pharmaceuticals, personal fees from Novartis, outside the submitted work; and Membership: AeDA; DGHNO; Deutsche Gesellschaft für Allergologie und klinische Immunologie; HNO-BV; GPA; EAACI.

E. Mélen has received consulting fees from ALK and AstraZeneca, lecture fees from ALK, AstraZeneca, Chiesi and Sanofi, outside the submitted study.

G. Koppelman reports grants from Netherlands Lung Foundation, grants from ZON MW (VICI grant), grants from H2020, grants from TEVA The Netherlands, grants from Vertex, grants from Netherlands Growth Fund (OMBION), personal fees from AZ, personal fees from PURE-IMS, personal fees from Sanofi, personal fees from Boehringer-Ingelheim, outside the submitted work; and Chair of the exquAIro foundation.

S. Toppila-Salmi reports other from ALK-Abelló, other from AstraZeneca, other from Clario, other from GSK, other from Sanofi, other from OrionPharma, grants from GSK, grants from Sanofi, outside the submitted work.

D. Larenas Linnemann reports personal fees from ALK, Armstrong, Astrazeneca national and global, Chiesi, GSK national and global, Megalabs Ecuador, Naos, Novartis, Pfizer, Sanofi, Siegfried, Syneos Health, grants from Abbvie, Lilly, Sanofi, Astrazeneca, Pfizer, Novartis, GSK, Chiesi, Biopharma, outside the submitted work; and Editor in chief of Immune System (Karger); Member of asthma committee ACAAI; Subgroup chair of allergen immunotherapy Practice parameter update JTF AAAAI/ACAAI 2024; Member of allergen immunotherapy committee AAAAI; Chair of allergen immunotherapy committee CMICA; Member of allergic asthma task force EAACI.T. Haahtela reports personal fees from ALK Nordic, outside the submitted work.

I. Ansotegui reports personal fees from Bayer, personal fees from Eurodrug, personal fees from Gebro, personal fees from Menarini, personal fees from MSD, personal fees from Roxall, personal fees from Sanofi, personal fees from Cipla, personal fees from Glenmark, personal fees from Opella, personal fees from GSK, personal fees from Stallergenes, outside the submitted work.

The other authors have nothing to declare, outside the submitted paper.
