# Supplementary material for: Distinction between allergic rhinitis alone and allergic rhinitis + asthma in MASK-air® real-world studies; a ARIA-WAO task force review
Source: World Allergy Organ J. 2026 Jul 31;19(8):101435. doi: 10.1016/j.waojou.2026.101435 (PMC13452204; doi:10.1016/j.waojou.2026.101435)
Supplement: Supplementary file 1 [file mmc1.docx]

**Table S1 online. Stepwise processes for understanding the differences between allergic rhinitis alone *versus* allergic rhinitis + asthma** (expanded from ^1^)

1- A clinical observation led to the concept that multimorbidity (allergic rhinitis [AR] + asthma [A]) and IgE polysensitisation may be linked. ^2^
2- Mechanistic ^3^ and epidemiologic studies (European Community Respiratory Health Survey: ECRHS, Framework Programme , FP2) ^4^ allowed us to better understand the links between asthma and AR. The concept of “one-airway-one-disease” ^5^ led to the ARIA guidelines. ^6^
3- AR alone affects around 70-80% of AR patients, whereas AR + A multimorbidity affects 20-30%. ^7^ On the other hand, most asthma patients have allergic or non-allergic rhinitis. ^8-10^ These data suggested common pathways between AR+A and rhinitis-specific pathways.
4- The severity of AR and A increases in parallel on exposure to an allergen. ^10^
5- The EU Network of Excellence GA^2^LEN (Global Allergy and Asthma European Network, FP6) ^11^ attempted to better understand sensitisation patterns. ^12^
6- MeDALL (Mechanisms of the Development of Allergy, FP7) ^13,14^ led to considerable improvement in understanding the mechanisms underlying the complex interactions between multimorbidity and polysensitisation (epidemiologic, genomic and epigenetic studies). ^12^
7- Overall, AR+A is associated with polysensitisation in Europe, ^15-25^, New Zealand, ^26^ Brazil ^27^ and China. ^28,29^
8- Moreover, by comparison to mono-sensitised patients, polysensitised ones have an earlier age of onset, more commonly an uncontrolled disease, higher eosinophil counts and total IgE levels ^30-32^ as well as different trajectories. ^16,33-35^
9- Impact of AR on A: Concomitant AR+A in asthma patients appears to contribute to increased asthma exacerbations and poorer asthma control, both in children ^36-40^ and adults globally. ^41-52^
10- MASK-air® (Mobile Airways Sentinel networK), an mHealth (mobile health) app, helped to capture direct patient-reported data to obtain novel insights into the complex interactions informed by MeDALL. ^53^
11- Clustering MASK-air® data using AR or asthma identified an extreme allergy phenotype including AR + asthma + conjunctivitis. This phenotype was more severe than single diseases alone. However, mHealth observational studies are only hypothesis generating. ^54^
12- Canonical epidemiologic studies showed that (i) the risk of adult-onset asthma increases with the number of allergic comorbidities, ^24^ (ii) severe asthma is associated with multimorbidity, ^55^ (iii) age of onset and parental allergy suggest that multimorbidity behaves differently to AR or asthma alone, ^30,31^ and (iv) the role of sex hormones at puberty appears to be influenced by multimorbidity.^56,57^
13- A discovery study in MeDALL (Europe, transcriptomics and RT-PCR) and a validation study in EVA-PR (Puerto-Rico, RNA sequencing) yielded the same results: Multimorbidity (AR+A+AD) was associated with 7 genes of T2 signalling: *IL-5* (eosinophils) and *IL-33* (polysensitisation and eosinophilia). ^58^ In MeDALL, AR-specific genes have been identified. These genes are mostly associated with TLR signalling pathways and *IL-17*. ^58,59^ There is almost no overlap between AR alone and AR+A. ^59^ In children and adolescents, no specific genes could be identified in asthma or atopic dermatitis alone. ^58^
14- There are shared epigenetic patterns of allergic multimorbidities, but, in the MeDALL study, in children, these epigenetic patterns were not found in asthma alone. ^60-62^ In nasal brushed cells in childhood, strong DNA-methylation signatures were shared by the AR+A phenotype, ^62^ confirming previous findings in blood.
15- An extreme allergy phenotype (AR+A + C or AR+A+ AD) is rare ^63-66^ but occurs more commonly than by chance. This extreme phenotype was confirmed by MASK-air^®^ and associated with lack of control and severity. ^67-69^
16- The new ARIA-MeDALL hypothesis was confirmed in a case control and an epidemiologic study in the general population (CONSTANCES). In CONSTANCES, participants with AR + asthma had more severe AR symptoms than those with AR alone^70,71^ as well as an earlier age of onset^71,72^. These results were replicated in the case control study EGEA (epidemiological study on the genetics and environment of asthma, bronchial hyperresponsiveness and atopy). ^73^
17- There are therapeutic consequences related to this hypothesis.

**References**

1.	Bousquet J, Melen E, Haahtela T, et al. Rhinitis associated with asthma is distinct from rhinitis alone: The ARIA-MeDALL hypothesis. *Allergy*. May 2023;78(5):1169–1203. doi:10.1111/all.15679

2.	Bousquet J, Coulomb Y, Arrendal H, Robinet-Levy M, Michel FB. Total serum IgE concentrations in adolescents and adults using the phadebas IgE PRIST technique. *Allergy*. 1982;37(6):397–406.

3.	Vignola AM, Chanez P, Godard P, Bousquet J. Relationships between rhinitis and asthma. *Allergy*. 1998;53(9):833–9.

4.	Leynaert B, Bousquet J, Neukirch C, Liard R, Neukirch F. Perennial rhinitis: An independent risk factor for asthma in nonatopic subjects: Results from the European Community Respiratory Health Survey. *J Allergy Clin Immunol*. 1999:301–304.

5.	Simons FE. Allergic rhinobronchitis: the asthma-allergic rhinitis link. *J Allergy Clin Immunol*. 1999;104(3 Pt 1):534–40.

6.	Bousquet J, Van Cauwenberge P, Khaltaev N. Allergic rhinitis and its impact on asthma. *J Allergy Clin Immunol*. 2001;108(5 Suppl):S147–334.

7.	Leynaert B, Neukirch C, Kony S, et al. Association between asthma and rhinitis according to atopic sensitization in a population-based study. *J Allergy Clin Immunol*. Jan 2004;113(1):86–93.

8.	Togias A, Gergen PJ, Hu JW, et al. Rhinitis in children and adolescents with asthma: Ubiquitous, difficult to control, and associated with asthma outcomes. *J Allergy Clin Immunol*. Mar 2019;143(3):1003–1011 e10. doi:10.1016/j.jaci.2018.07.041

9.	Cruz AA, Popov T, Pawankar R, et al. Common characteristics of upper and lower airways in rhinitis and asthma: ARIA update, in collaboration with GA(2)LEN. *Allergy*. 2007;62 Suppl 84:1–41. doi:ALL1551 [pii]

10.1111/j.1398-9995.2007.01551.x

10.	Togias AG. Systemic immunologic and inflammatory aspects of allergic rhinitis. *J Allergy Clin Immunol*. Nov 2000;106(5 Suppl):S247–50.

11.	Bousquet J, Burney PG, Zuberbier T, et al. GA2LEN (Global Allergy and Asthma European Network) addresses the allergy and asthma 'epidemic'. *Allergy*. Jul 2009;64(7):969–77. doi:10.1111/j.1398-9995.2009.02059.x

12.	Bousquet J, Anto JM, Bachert C, et al. Factors responsible for differences between asymptomatic subjects and patients presenting an IgE sensitization to allergens. A GALEN project. *Allergy*. Jun 2006;61(6):671–80.

13.	Bousquet J, Anto J, Auffray C, et al. MeDALL (Mechanisms of the Development of ALLergy): an integrated approach from phenotypes to systems medicine. *Allergy*. May 2011;66(5):596–604. doi:10.1111/j.1398-9995.2010.02534.x

14.	Anto JM, Bousquet J, Akdis M, et al. Mechanisms of the Development of Allergy (MeDALL): Introducing novel concepts in allergy phenotypes. *J Allergy Clin Immunol*. Feb 2017;139(2):388–399. doi:10.1016/j.jaci.2016.12.940

15.	Westman M, Lupinek C, Bousquet J, et al. Early childhood IgE reactivity to pathogenesis-related class 10 proteins predicts allergic rhinitis in adolescence. *J Allergy Clin Immunol*. May 2015;135(5):1199–206 e1–11. doi:10.1016/j.jaci.2014.10.042

16.	Asarnoj A, Hamsten C, Waden K, et al. Sensitization to cat and dog allergen molecules in childhood and prediction of symptoms of cat and dog allergy in adolescence: A BAMSE/MeDALL study. *J Allergy Clin Immunol*. Mar 2016;137(3):813–21 e7. doi:10.1016/j.jaci.2015.09.052

17.	Mikkelsen S, Dinh KM, Boldsen JK, et al. Combinations of self-reported rhinitis, conjunctivitis, and asthma predicts IgE sensitization in more than 25,000 Danes. *Clin Transl Allergy*. Mar 2021;11(1):e12013. doi:10.1002/clt2.12013

18.	Filiou A, Holmdahl I, Asarnoj A, et al. Development of Sensitization to Multiple Allergen Molecules from Preschool to School Age Is Related to Asthma. *Int Arch Allergy Immunol*. Jan 18 2022:1–12. doi:10.1159/000521324

19.	Blondal V, Malinovschi A, Sundbom F, et al. Multimorbidity in asthma, association with allergy, inflammatory markers and symptom burden, results from the Swedish GA(2) LEN study. *Clin Exp Allergy*. Feb 2021;51(2):262–272. doi:10.1111/cea.13759

20.	Schoos AM, Jelding-Dannemand E, Stokholm J, Bonnelykke K, Bisgaard H, Chawes BL. Single and multiple time-point allergic sensitization during childhood and risk of asthma by age 13. *Pediatr Allergy Immunol*. Nov 2019;30(7):716–723. doi:10.1111/pai.13109

21.	Raciborski F, Bousquet J, Bousqet J, et al. Dissociating polysensitization and multimorbidity in children and adults from a Polish general population cohort. *Clin Transl Allergy*. 2019;9:4. doi:10.1186/s13601-019-0246-y

22.	Schmidt F, Hose AJ, Mueller-Rompa S, et al. Development of atopic sensitization in Finnish and Estonian children: A latent class analysis in a multicenter cohort. *J Allergy Clin Immunol*. May 2019;143(5):1904–1913 e9. doi:10.1016/j.jaci.2018.12.1014

23.	Hose AJ, Depner M, Illi S, et al. Latent class analysis reveals clinically relevant atopy phenotypes in 2 birth cohorts. *J Allergy Clin Immunol*. Jun 2017;139(6):1935–1945 e12. doi:10.1016/j.jaci.2016.08.046

24.	Toppila-Salmi S, Chanoine S, Karjalainen J, Pekkanen J, Bousquet J, Siroux V. Risk of adult-onset asthma increases with the number of allergic multimorbidities and decreases with age. *Allergy*. Dec 2019;74(12):2406–2416. doi:10.1111/all.13971

25.	Bengtsson C, Lindberg E, Jonsson L, et al. Chronic Rhinosinusitis Impairs Sleep Quality: Results of the GA2LEN Study. *Sleep*. Jan 1 2017;40(1)doi:10.1093/sleep/zsw021

26.	Sears MR, Burrows B, Flannery EM, Herbison GP, Holdaway MD. Atopy in childhood. I. Gender and allergen related risks for development of hay fever and asthma. *Clin Exp Allergy*. Nov 1993;23(11):941–8.

27.	Aranda CS, Cocco RR, Pierotti FF, et al. Allergic sensitization pattern of patients in Brazil. *J Pediatr (Rio J)*. Jul–Aug 2021;97(4):387–395. doi:10.1016/j.jped.2020.08.005

28.	Zhang W, Xie B, Liu M, Wang Y. Associations between sensitisation to allergens and allergic diseases: a hospital-based case-control study in China. *BMJ open*. Feb 1 2022;12(2):e050047. doi:10.1136/bmjopen-2021-050047

29.	Gao Z, Fu WY, Sun Y, et al. Artemisia pollen allergy in China: Component-resolved diagnosis reveals allergic asthma patients have significant multiple allergen sensitization. *Allergy*. Feb 2019;74(2):284–293. doi:10.1111/all.13597

30.	Burte E, Bousquet J, Siroux V, Just J, Jacquemin B, Nadif R. The sensitization pattern differs according to rhinitis and asthma multimorbidity in adults: the EGEA study. *Clin Exp Allergy*. Feb 25 2017;doi:10.1111/cea.12897

31.	Siroux V, Ballardini N, Soler M, et al. The asthma-rhinitis multimorbidity is associated with IgE polysensitization in adolescents and adults. *Allergy*. Jul 2018;73(7):1447–1458. doi:10.1111/all.13410

32.	Kauffmann F, Dizier MH, Annesi-Maesano I, et al. EGEA (Epidemiological study on the Genetics and Environment of Asthma, bronchial hyperresponsiveness and atopy)-- descriptive characteristics. *Clin Exp Allergy*. Dec 1999;29 Suppl 4:17–21.

33.	Asarnoj A, Hamsten C, Lupinek C, et al. Prediction of peanut allergy in adolescence by early childhood storage protein-specific IgE signatures: The BAMSE population-based birth cohort. *J Allergy Clin Immunol*. Feb 09 2017;doi:10.1016/j.jaci.2016.12.973

34.	Ballardini N, Bergstrom A, Wahlgren CF, et al. IgE antibodies in relation to prevalence and multimorbidity of eczema, asthma, and rhinitis from birth to adolescence. *Allergy*. Mar 2016;71(3):342–9. doi:10.1111/all.12798

35.	Wickman M, Lupinek C, Andersson N, et al. Detection of IgE Reactivity to a Handful of Allergen Molecules in Early Childhood Predicts Respiratory Allergy in Adolescence. *EBioMedicine*. Dec 2017;26:91–99. doi:10.1016/j.ebiom.2017.11.009

36.	Kang HY, Park CS, Bang HR, Sazonov V, Kim CJ. Effect of allergic rhinitis on the use and cost of health services by children with asthma. *Yonsei Med J*. Aug 30 2008;49(4):521–9. doi:10.3349/ymj.2008.49.4.521

37.	de Groot EP, Nijkamp A, Duiverman EJ, Brand PL. Allergic rhinitis is associated with poor asthma control in children with asthma. *Thorax*. Jul 2012;67(7):582–7. doi:10.1136/thoraxjnl-2011-201168

38.	Padilla J, Uceda M, Ziegler O, Lindo F, Herrera-Perez E, Huicho L. Association between allergic rhinitis and asthma control in Peruvian school children: a cross-sectional study. *Biomed Res Int*. 2013;2013:861213. doi:10.1155/2013/861213

39.	Pongracic JA, Krouse RZ, Babineau DC, et al. Distinguishing characteristics of difficult-to-control asthma in inner-city children and adolescents. *J Allergy Clin Immunol*. Oct 2016;138(4):1030–1041. doi:10.1016/j.jaci.2016.06.059

40.	Butz AM, Bellin M, Tsoukleris M, et al. Very Poorly Controlled Asthma in Urban Minority Children: Lessons Learned. *J Allergy Clin Immunol Pract*. May–Jun 2018;6(3):844–852. doi:10.1016/j.jaip.2017.08.007

41.	Halpern MT, Schmier JK, Richner R, Guo C, Togias A. Allergic rhinitis: a potential cause of increased asthma medication use, costs, and morbidity. *J Asthma*. Feb 2004;41(1):117–26.

42.	Bousquet J, Gaugris S, Kocevar VS, et al. Increased risk of asthma attacks and emergency visits among asthma patients with allergic rhinitis: a subgroup analysis of the improving asthma control trial. *Clin Exp Allergy*. Jun 2005;35(6):723–7.

43.	Gaugris S, Sazonov-Kocevar V, Thomas M. Burden of concomitant allergic rhinitis in adults with asthma. *J Asthma*. Feb 2006;43(1):1–7.

44.	Ponte EV, Franco R, Nascimento HF, et al. Lack of control of severe asthma is associated with co-existence of moderate-to-severe rhinitis. *Allergy*. May 2008;63(5):564–9. doi:10.1111/j.1398-9995.2007.01624.x

45.	Magnan A, Meunier JP, Saugnac C, Gasteau J, Neukirch F. Frequency and impact of allergic rhinitis in asthma patients in everyday general medical practice: a French observational cross-sectional study. *Allergy*. Mar 2008;63(3):292–8. doi:10.1111/j.1398-9995.2007.01584.x

46.	Vandenplas O, Dramaix M, Joos G, et al. The impact of concomitant rhinitis on asthma-related quality of life and asthma control. *Allergy*. Oct 2010;65(10):1290–7. doi:10.1111/j.1398-9995.2010.02365.x

47.	Ohta K, Bousquet PJ, Aizawa H, et al. Prevalence and impact of rhinitis in asthma. SACRA, a cross-sectional nation-wide study in Japan. Research Support, Non-U.S. Gov't. *Allergy*. Oct 2011;66(10):1287–95. doi:10.1111/j.1398-9995.2011.02676.x

48.	Bin Mahfouz T, Banjar SA, Assiri RA, Alshehri GA, Binyousef F. The Prevalence and Impact of Allergic Rhinitis on Asthma Exacerbations in Asthmatic Adult Patients in the Riyadh Region of Saudi Arabia: A Cross-Sectional Study. *Cureus*. Dec 2022;14(12):e32324. doi:10.7759/cureus.32324

49.	Megersa S, Chala G, Fikremariam K. Determinants of Asthma Attack Among Adult Asthmatic Patients Attending at Public Hospitals of West Shoa Zone, Oromia Regional State, Ethiopia, 2021: Case-Control Study. *J Asthma Allergy*. 2022;15:1143–1154. doi:10.2147/JAA.S376499

50.	Geleta LA, Dadi LS, Sona AA. Determinants of uncontrolled asthma among adult asthmatic patients on follow-up at chest clinic of Jimma medical center: unmatched case-control study. *J Asthma*. Jun 2022;59(6):1103–1109. doi:10.1080/02770903.2021.1908351

51.	Sriprasart T, Saiphoklang N, Kawamatawong T, et al. Allergic rhinitis and other comorbidities associated with asthma control in Thailand. *Front Med (Lausanne)*. 2023;10:1308390. doi:10.3389/fmed.2023.1308390

52.	Scelo G, Torres-Duque CA, Maspero J, et al. Analysis of comorbidities and multimorbidity in adult patients in the International Severe Asthma Registry. *Ann Allergy Asthma Immunol*. Jan 2024;132(1):42–53. doi:10.1016/j.anai.2023.08.021

53.	Bousquet J, Bedbrook A, Czarlewski W, et al. Guidance to 2018 good practice: ARIA digitally-enabled, integrated, person-centred care for rhinitis and asthma. *Clin Transl Allergy*. 2019;9:16. doi:10.1186/s13601-019-0252-0

54.	Bousquet J, Anto JM, Bachert C, et al. ARIA digital anamorphosis: Digital transformation of health and care in airway diseases from research to practice. *Allergy*. Jan 2021;76(1):168–190. doi:10.1111/all.14422

55.	Toppila-Salmi S, Lemmetyinen R, Chanoine S, et al. Risk factors for severe adult-onset asthma: a multi-factor approach. *BMC Pulm Med*. Jul 8 2021;21(1):214. doi:10.1186/s12890-021-01578-4

56.	Frohlich M, Pinart M, Keller T, et al. Is there a sex-shift in prevalence of allergic rhinitis and comorbid asthma from childhood to adulthood? A meta-analysis. *Clin Transl Allergy*. 2017;7:44. doi:10.1186/s13601-017-0176-5

57.	Rosario CS, Cardozo CA, Neto HJC, Filho NAR. Do gender and puberty influence allergic diseases? *Allergol Immunopathol (Madr)*. 2021;49(2):122–125. doi:10.15586/aei.v49i2.49

58.	Lemonnier N, Melen E, Jiang Y, et al. A novel whole blood gene expression signature for asthma, dermatitis, and rhinitis multimorbidity in children and adolescents. *Allergy*. Apr 11 2020;75:3248–60. doi:10.1111/all.14314

59.	Aguilar D, Lemonnier N, Melen E, et al. Distinction between rhinitis alone and rhinitis with asthma using interactomics. *Sci Rep*. Aug 12 2023;13(1):13125. doi:10.1038/s41598-023-39987-6

60.	Xu CJ, Soderhall C, Bustamante M, et al. DNA methylation in childhood asthma: an epigenome-wide meta-analysis. *Lancet Respir Med*. May 2018;6(5):379–388. doi:10.1016/S2213-2600(18)30052-3

61.	Xu CJ, Gruzieva O, Qi C, et al. Shared DNA methylation signatures in childhood allergy: The MeDALL study. *J Allergy Clin Immunol*. Mar 2021;147(3):1031–1040. doi:10.1016/j.jaci.2020.11.044

62.	Qi C, Jiang Y, Yang IV, et al. Nasal DNA methylation profiling of asthma and rhinitis. *J Allergy Clin Immunol*. Jun 2020;145(6):1655–1663. doi:10.1016/j.jaci.2019.12.911

63.	Pinart M, Benet M, Annesi-Maesano I, et al. Comorbidity of eczema, rhinitis, and asthma in IgE-sensitised and non-IgE-sensitised children in MeDALL: a population-based cohort study. *Lancet Respir Med*. Feb 2014;2(2):131–40. doi:10.1016/S2213-2600(13)70277-7

64.	Garcia-Aymerich J, Benet M, Saeys Y, et al. Phenotyping asthma, rhinitis and eczema in MeDALL population-based birth cohorts: an allergic comorbidity cluster. *Allergy*. Aug 2015;70(8):973–84. doi:10.1111/all.12640

65.	Pols DHJ, Nielen MMJ, Korevaar JC, Bindels PJE, Bohnen AM. Reliably estimating prevalences of atopic children: an epidemiological study in an extensive and representative primary care database. *NPJ Prim Care Respir Med*. Apr 13 2017;27(1):23. doi:10.1038/s41533-017-0025-y

66.	Pols DH, Wartna JB, van Alphen EI, et al. Interrelationships between Atopic Disorders in Children: A Meta-Analysis Based on ISAAC Questionnaires. *PloS one*. 2015;10(7):e0131869. doi:10.1371/journal.pone.0131869

67.	Bousquet J, Devillier P, Anto JM, et al. Daily allergic multimorbidity in rhinitis using mobile technology: A novel concept of the MASK study. *Allergy*. Aug 2018;73(8):1622–1631. doi:10.1111/all.13448

68.	Bousquet J, Sousa-Pinto B, Anto JM, et al. Identification by cluster analysis of patients with asthma and nasal symptoms using the MASK-air(R) mHealth app. *Pulmonology*. Nov 22 2022;doi:10.1016/j.pulmoe.2022.10.005

69.	Sousa-Pinto B, Schunemann HJ, Sa-Sousa A, et al. Consistent trajectories of rhinitis control and treatment in 16,177 weeks: The MASK-air(R) longitudinal study. *Allergy*. Apr 2023;78(4):968–983. doi:10.1111/all.15574

70.	Savoure M, Bousquet J, Leynaert B, et al. Rhinitis phenotypes and multimorbidities in the general population: the CONSTANCES cohort. *Eur Respir J*. Feb 2023;61(2)doi:10.1183/13993003.00943-2022

71.	Savoure M, Bousquet J, Leynaert B, et al. Rhinitis phenotypes and multimorbidities in the general population Constances cohort. *Eur Respir J*. Oct 6 2022;doi:10.1183/13993003.00943-2022

72.	Burte E, Bousquet J, Siroux V, Just J, Jacquemin B, Nadif R. The sensitization pattern differs according to rhinitis and asthma multimorbidity in adults: the EGEA study. *Clin Exp Allergy*. Feb 25 2017;47(4):520–529. doi:10.1111/cea.12897

73.	Burte E, Bousquet J, Varraso R, et al. Characterization of Rhinitis According to the Asthma Status in Adults Using an Unsupervised Approach in the EGEA Study. *PloS one*. 2015;10(8):e0136191. doi:10.1371/journal.pone.0136191
